# Influence of drought stress and mycorrhizal (*Funneliformis mosseae*) symbiosis on growth parameters, chlorophyll fluorescence, antioxidant activity, and essential oil composition of summer savory (*Satureja hortensis* L.) plants

**DOI:** 10.3389/fpls.2023.1151467

**Published:** 2023-06-05

**Authors:** Farzad Rasouli, Trifa Amini, Sona Skrovankova, Mohammad Asadi, Mohammad Bagher Hassanpouraghdam, Sezai Ercisli, Martina Buckova, Martina Mrazkova, Jiri Mlcek

**Affiliations:** ^1^ Department of Horticulture, Faculty of Agriculture, University of Maragheh, Maragheh, Iran; ^2^ Department of Food Analysis and Chemistry, Faculty of Technology, Tomas Bata University in Zlin, Zlin, Czechia; ^3^ Department of Plant Production and Genetics, Faculty of Agriculture, University of Maragheh, Maragheh, Iran; ^4^ Department of Horticulture, Faculty of Agriculture, Ataturk University, Erzurum, Türkiye; ^5^ HGF Agro, Ata Teknokent, Erzurum, Türkiye

**Keywords:** summer savory (*Satureja hortensis* L.), drought stress, arbuscular mycorrhiza fungi (AMF), growth parameters, chlorophyll fluorescence, antioxidant activity, essential oil

## Abstract

**Introduction:**

Drought stress unfavorably influences the growth and physiological traits of plants in the arid and semi-arid regions of the world. This study aimed to determine the effects of arbuscular mycorrhiza fungi (AMF; *Funneliformis mosseae*) inoculation on the physiological and biochemical responses of summer savory (*Satureja hortensis* L.) under different irrigation regimes.

**Methods:**

The first factor was different irrigation regimes, including no drought stress (100% field capacity; FC), moderate drought stress (60% FC), and severe drought stress (30% FC); the second factor included the plants without AMF (AMF_0_) and with AMF inoculation (AMF_1_).

**Results:**

The results showed that better values, higher plant height, shoot mass (fresh and dry weight), relative water content (RWC), membrane stability index (MSI), photosynthesis pigments, *Fv*, *Fm*, *Fv*/*Fm*, and total soluble proteins were obtained in the plants inoculated with AMF. The highest values were obtained for plants with no drought stress, then the plants subjected to AMF_1_ under 60% FC, and the lowest ones for plants under 30% FC without AMF inoculation. Thus, these properties are reduced under moderate and severe drought stress. At the same time, the utmost activity of superoxide dismutase (SOD), ascorbate peroxidase (APX), guaiacol peroxidase (GPX), and the highest malondialdehyde (MDA), H_2_O_2_, proline, and antioxidant activity (TAA) were achieved for 30% FC + AMF_0_. It was also found that AMF inoculation improved essential oil (EO) composition, also as EO obtained from plants under drought stress. Carvacrol (50.84-60.03%) was the dominant component in EO; γ-terpinene (19.03-27.33%), *p*-cymene, α-terpinene, and myrcene, were recognized as other important components in EO. The higher carvacrol and γ-terpinene contents were obtained from summer savory plants with AMF inoculation and the lowest for plants without AMF and under 30% FC.

**Conclusion:**

According to the present findings, using AMF inoculation could be a sustainable and eco-friendly approach to improve physiological and biochemical characteristics and the essential oil quality of summer savory plants under water shortage conditions.

## 1 Introduction

Summer savory (*Satureja hortensis* L.) is an annual Lamiaceae family plant with aromatic qualities and several medicinal properties related to its antioxidant, antifungal, antibacterial, and antiseptic characteristics (Fierascu et al., 2018). The herb is tolerant to different weather conditions and based on previous findings, the summer savory plant can be introduced into agricultural systems as a valuable drought-tolerant medicinal plant, especially in areas with low rainfall and scarce water resources (Karimi et al., 2022). Therefore, farmers focused on expanding summer savory plant cultivation in water-scarce conditions, while protecting the natural biodiversity and adequate productivity (Jaleel et al., 2007).

Drought stress is one of the most important abiotic stresses that adversely affect the physiological and biochemical characteristics and the growth and development of plants in the arid and semi-arid regions of the world (Kafi et al., 2010). The impact of drought stress on the production of secondary metabolites is related to the plant’s physiological state during stress, leading to the accumulation of reactive oxygen species (ROS) in the plants (Dossa et al., 2017). Various stresses, including drought, have been shown to affect metabolic pathways in the *Lamiaceae* family that are highly effective in producing terpenes and phenolics (Shariat et al., 2016). Alhaithloul et al. (Alhaithloul et al., 2019) reported that drought stress caused an increase in the level of tannins, terpenoids, and alkaloids in mint plants. In sesame, an increase in the concentration of carotenoids under drought conditions has been reported (Yousefzadeh Najafabadi and Ehsanzadeh, 2017). To prevent or reduce ROS damage, plants promote enzymatic antioxidants such as ascorbate peroxidase (APX), superoxide dismutase (SOD), catalase (CAT), and peroxidase (POD), and non-enzymatic antioxidants as carotenoids, flavonoids, phenols, anthocyanins, and some free amino acids (Agarwal and Pandey, 2004; Hanin et al., 2016).

There are several strategies for bearing the drought stress for plants, such as the use of native microorganisms. The strategy could include beneficial microbes that directly or indirectly enhance tolerance mechanisms or appropriate mineral fertilization. In sustainable agricultural systems, using arbuscular mycorrhizae fungi (AMF), especially in poor soils, can help to increase crop productivity and improve soil quality (Kyriazopoulos et al., 2014). AMF is a valuable essential microorganism that grows in the soil and can establish a symbiotic relationship with nearly 90% of plant species, including bryophytes, ferns, and flowering plants (Ahanger et al., 2014; Begum et al., 2019). AMF symbiosis can cause better plant growth, more absorption of nutrients, more tolerance against abiotic and biotic stresses, and improvement of soil structure in the host plant (Wu et al., 2013). Studies (Wu et al., 2013; Balliu et al., 2015; Hazzoumi et al., 2015) have shown that plants colonized with AMF showed higher enzyme activity, mineral nutrition content, and photosynthesis potential.

AMF inoculation is a relevant method to stimulate plant growth and enhance essential oils biosynthesis in aromatic and medicinal plants (Rydlová et al., 2016). Hazzoumi et al. (Hazzoumi et al., 2015) reported that basil plants inoculated by mycorrhiza showed higher total chlorophyll content than non-inoculated plants. In lettuce, the application of AMF caused a significant increase in the growth and development of the plant (Asadi et al., 2022). It has also been reported that indigenous microorganisms of arid and semi-arid regions have the advantage of drought tolerance that can live and help host plants to withstand drought stress (Shirinbayan et al., 2019). Under drought stress, AMF inoculation improves plant growth by increasing root length, leaf area, plant biomass, nutrient uptake, and soil structure (Rabab et al., 2016). AMF hyphae penetrate the plant roots, grow extensively between the living cells of the root cortex, and form a substantial and dynamic relationship between the symbionts (plant and fungus). As a result, this colonization increases growth and yield and reduces the effects of stress (Mathur et al., 2018). Thus, AMF increases the absorption of phosphorus, zinc, copper, and potassium. Therefore, a symbiosis occurs between the plant and the fungus, which significantly improves the plant’s physiology and nutritional conditions and mitigates the adverse effects of drought stress (Augé, 2001; Wężowicz et al., 2017). Some other phenomena that are amplified by AMF include changes in plant hormone balance, water absorption through hydraulic conduction of external hyphae, osmotic regulation [25], increased antioxidant activity, increased nutrient absorption, and increased photosynthesis [26]. Previous studies have shown that the AMF-inoculated plants achieve increased essential oil (EO) quantity and quality in plants such as thyme (*Thymus vulgaris* L.) (Amani Machiani et al., 2021), peppermint (*Mentha* x *piperita* L.) (Ostadi et al., 2020), and holy basil (*Ocimum tenuiflorum* L.) (Thokchom et al., 2020).

The interaction of drought stress and AMF inoculation in terms of essential oil profile has not been previously investigated for summer savory, while these factors were studied separately on the summer savory. The current research is presented about the hypothesis that the morphological and physiological properties of summer savory plants and the content and composition of essential oil are affected by drought stress. We then hypothesize that AMF inoculation reduces the effects of drought stress. This study, therefore, aims to investigate the inoculation of AMF on ameliorating the effect of drought stress on the summer savory plant and also to evaluate the impact of AMF on some morphological and biochemical responses of the plant.

## 2 Materials and methods

The current research was conducted as a factorial experiment based on a completely randomized design (CRD) with four replications, so that each plant was considered as a replication. The experiment was performed in the research greenhouse of the Department of Horticultural Sciences of Maragheh University in East Azarbaijan province with the geographical coordinates of 37° 23′ north latitude and 46°16′ east longitude and an altitude of 1485 meters above sea level. The night and day temperatures were 17-23°C, respectively, and the relative humidity was about 65-75% in the greenhouse. The study was conducted as a factorial experiment based on the completely randomized design with five replications. The experimental treatments contained two factors; the first factor included three irrigation regimes, regular irrigation (100% filed capacity (FC)), moderate (60% FC), and severe (30% FC) drought stress; the second factor was the treatment without AMF (*Funneliformis mosseae*) and inoculation with AMF (5 g kg^-1^ soil).

The savory seeds used in this experiment were purchased from the Pakan Bazr company, in Isfahan, Iran. Seeds were sown in a plastic pot (5 L volume), and the soil mix was 1:1:2; sand: animal manure: topsoil, respectively which was sandy clay loam containing 0.96% organic carbon, 0.08% total N, 10.13 and 493.25 mg kg^-1^ soil of available P and K, respectively, and the pH was 7.96. After seeds germination, pots were watered by graduated cylinder daily to 100% FC for two weeks. The FC was calculated by the gravimetric method. Maintenance of the drought treatments was attained by daily weighing, and the water lost in pots was replaced using a precision scale (× 0.1 accuracies). These water deficit treatments were employed for two months until harvest.

### 2.1 Mycorrhizal root colonization

Colonization of the savory plant roots was investigated using the crossover method (Giovannetti and Mosse, 1980). To color the roots, after cleaning and separating from the soil particles, the roots were divided into small pieces (1 cm) and placed inside the KOH solution (10%) for 10 min. After washing with distilled water, the parts were acidified with HCl (2%; v/v) for 20 min. Later, staining was done with trypan blue (0.05%) and lactic acid (80%; v/v) for 12 hours. The stained roots were washed with distilled water and kept in a solution containing water, glycerol, and lactic acid (1:1:1; v/v/v). The Olympus microscope (BH-2) discriminated and assessed the stained root pieces. The blue AMF organs and hyphae were recorded as well-sharped photos.

### 2.2 Measurement of morphological characteristics

The shoot mass of fresh weight (FW) was measured immediately after plant harvest. Then, the shoots were dried in an oven at 75°C for 48 hours, and their dry weight (DW) was recorded using a digital scale (A&D weighing, Japan, accuracy 0.01 mg). The height of the plants was also measured using a digital caliper.

### 2.3 Estimation of membrane stability index

To assay, the MSI, 200 mg of leaf sample was placed in 10 cubic centimeters of deionized water in a water bath at 40°C for 30 min, and then the EC1 (Electrical conductivity) was measured. Also, the second set of samples was boiled at 100°C in a water bath for 10 min, and its EC2 was measured. Finally, the percentage of MSI was obtained from the following formula of Sairam (Sairam et al., 2002):


$$MSI\;\left(\%\right)=1-\frac{EC1}{EC2}\;\times 100$$

### 2.4 Determination of relative water content

To estimate RWC, leaf pieces (1 cm^2^) were weighed (FW) and immediately floated in Petri dishes containing deionized water. After being placed in the darkness for 24 hours, the samples were saturated with water and weighed (TW). The dry weight of the parts was recorded after drying at 70°C for 48 hours (DW). The amount of RWC was calculated as the following formula (Hayat et al., 2007):


$$RWC\;\left(\%\right)=\frac{\left(FW-DW\right)}{TW-DW}\times 100$$

### 2.5 Determination of hydrogen peroxide content

The content of H_2_O_2_ was measured based on the method of Alexieva et al. (Alexieva et al., 2001). Briefly, 50 mg of fresh young leaves were digested with 5 ml of 0.1% TCA. After centrifugation of the homogeneous mixture at 4°C and at 12000 rpm for 15 min, 500 μl of the supernatant was mixed with 500 μl of potassium phosphate buffer (100 mM) and 2 ml of potassium iodide (KI) solution. Then, the reaction mixture was incubated for 60 minutes in the darkness at room temperature, and the absorbance was read at 390 nm (UV-1800, Shimadzu, Tokyo, Japan).

### 2.6 Measurement of malondialdehyde content

The MDA content as a measure of lipid peroxidation was obtained according to the method of Dhindsa et al. (Dhindsa et al., 1981). The leaf (0.5 g) sample was homogenized by adding 5 ml of 0.1% trichloroacetic acid (TCA). After centrifugation (10000 rpm at 4°C for 15 min), the supernatant (300 μL) was mixed with 1 ml of 0.5% thiobarbituric acid (TBA), and the mixture was heated at 95°C for 30 min, and then the reaction solution was cooled on ice to stop the reaction. After centrifugation at 10000 rpm for 10 min, the absorbance was recorded at 440, 532, and 600 nm wavelengths.

### 2.7 Determination of proline content

To measure proline content (Bates et al., 1973), 0.5 g of leaf fresh samples were extracted with 10 ml of 3% sulfosalicylic acid on an ice bath to prevent proline degradation. Then, the extracts were centrifuged at 10000 rpm for 20 min at 4°C. Afterward, 2 ml of the supernatant was separated and mixed into glass tubes along with 2 ml of ninhydrin acid and 2 ml of glacial acetic acid and heated in a hot water bath, and immediately transferred to an ice bath for one hour. Later, 4 ml of toluene was added to the mixture and vortexed for 20 s. The absorbance was read using a spectrophotometer at 520 nm (nanometer).

### 2.8 Total soluble proteins content

Total protein content was determined following Bradford (Bradford, 1976), with bovine serum albumin as a standard curve with the solutions containing 0, 0.2, 0.4, 0.6, 0.8, or 1 mg mL^-1^ to which 100 μL of the Bradford solution was added. To prepare, the Bradford reagent 50 mg of coomassie brilliant blue G-250 dissolved in methanol (50 mL) and phosphoric acid (100 mL, 85%; w/v) and then was mixed with 850 mL distilled water and filtered. Finally, 1000 μL of the Bradford reagent was added to 50 μL the extract and incubated for 5 min at room temperature. Absorbance was recorded at 595 nm with a spectrophotometer.

### 2.9 Measurement of total antioxidant activity

Total antioxidant activity by the ferric-reducing ability of plasma (FRAP) method was done based on the protocol of Benzie et al. (Benzie and Strain, 1996). 0.1 M acetate buffer solution (pH 3.6), 10 mM TPTZ [2,4,6-tris (2-pyridyl) - 1,3,5-triazine] were mixed (10:1:1; v/v/v) as a FRAP reagent. 200 μl of summer savory plant extract was combined with 2950 µl of FRAP reagent and then kept at 30 min. Finally, the reaction mixture was read at 593 nm using a spectrophotometer.

### 2.10 Assay of guaiacol peroxidase activity

To prepare a plant extract for the activity of enzymes and protein content, 3 ml of 50 mM potassium phosphate buffer was added to 0.5 g of leaf fresh weight (pH 7) and vortexed. The extract also included 1 mM EDTA and 1% polyvinylpyrrolidone (PVP; w/v). The resulting extract was centrifuged at 13000 rpm for 15 min. The supernatant was used to measure protein content and enzyme activities.

The activity of the GPX enzyme was measured by the Cakmak et al. (Cakmak et al., 2001) method. The reaction mixture consisted of 750 μL of 100 mM potassium phosphate buffer (pH 7), 100 μL of H_2_O_2_ (70 mM), 750 μL of guaiacol (10 mM), and the extract. GPX enzyme activity was measured at 470 nm. The extinction coefficient equivalent to 26.6 mM^-1^ cm^-1^ was considered in the calculation of enzyme activity.

### 2.11 Assay of ascorbate peroxidase activity

APX activity was measured by the method of Chen and Asada (Chen and Asada, 1992). The reaction mixture consisted of 200 μl of ascorbate (2 mM), 750 μl of potassium phosphate buffer (100 mM), 200 μl of hydrogen peroxide (2 mM), and the leaf extract. The reaction was started by adding H_2_O_2_ to the desired mixture, and the APX enzyme activity was measured by reducing the absorbance at 290 nm with a spectrophotometer due to the consumption of H_2_O_2_. The extinction coefficient of 2.8 mM^-1^ cm^-1^ was also included in the calculation of APX enzyme activity.

### 2.12 Assay of superoxide dismutase activity

The SOD activity was measured according to the modified method of Fu et al. (Fu and Huang, 2001). 20 μl of the enzyme extract was added to 63 μM nitrotetrazolium blue (NBT), 1.3 μM riboflavin, 13 mM methionine, and 0.1 mM EDTA in 50 mM phosphate buffer (pH 7.8). Test tubes containing each reaction mixture were placed under incandescent light for 10 minutes and absorbance was recorded at 560 nm with a spectrophotometer.

### 2.13 Chlorophyll fluorescence parameters

Chlorophyll fluorescence parameters were including *F_0_* (minimum amount of chlorophyll fluorescence), (*Fm*) the maximum amount of chlorophyll fluorescence, (*Fv*) difference between *F_0_* and *Fm*, (*Fv*/*Fm*) maximum PSII quantum efficiency and also, and Y (NO) non-photochemical PSII quantum efficiency. Four developed and relatively young leaves were randomly selected to measure the chlorophyll fluorescence parameters. The measurement of the mentioned parameter was performed using a pulse amplitude modulation fluorometer (PAM-2500, Heinz Walls, and Effeltrich, Germany). After the plants were adapted to the dark, the data were analyzed by PamWin software.

### 2.14 Photosynthetic pigments content

The content of photosynthetic pigments; chlorophyll a (Chl *a*), chlorophyll b (Chl *b*), and carotenoids (CARs) in the young developed leaves of the savory plant was determined by the method of Arnon (Arnon, 1949). Doing this, 0.5 g of fresh leaves was extracted by 10 ml of acetone (80%) and then centrifuged at 12000 rpm for 10 min. Lastly, the absorbance was read at 663, 645, and 470 nm, respectively, using a spectrophotometer (UV-1800, Shimadzu, Tokyo, Japan). The following formulas were used to calculate the content of Chl *a*, Chl *b*, and CARs:


(1) $$\text{Chl }a=\left[12.7\left({\text{A}}_{663}\right)-2.69\left({\text{A}}_{645}\right)\right]$$

(2) $$\text{Chl }b=\left[21.50\left({\text{A}}_{645}\right)-5.10\left({\text{A}}_{663}\right)\right]$$

(3) $$\text{CARs}=\left[1000\left({\text{A}}_{470}\right)-1.82\text{ Chl }a-85.02\text{ Chl }b\right]/198$$

### 2.15 Essential oil extraction and GC-MS analysis

Essential oil of the dried summer savory parts was isolated by distillation with water using a Clevenger-type apparatus. The amount of essential oil was measured using a calibrated burette, and the oils were stored at 5°C until analysis by gas chromatography. The GC instrument used was an Agilent 7890A (Santa Clara, CA, USA) equipped with a mass selective detector and an HP5 MS column (column length 30 m, inner diameter 0.25 lm, film thickness 0.25 mm) (voltage 70 eV). Helium was used as carrier gas with a flow rate of 0.8 ml/min. The column temperature was 280°C for the initial and the final injector temperature was 300°C. The column temperature is programmed at a rate of 4°C/min. The separation ratio was set to 40:1. 1 μL of each sample was injected into the GC with the injector in split mode (split ratio 40:1). A mixture of aliphatic hydrocarbons (C8-C40) was injected into the GC system under the same analytical conditions to calculate and identify the peaks. The compounds of the extracted essential oils were identified by comparing their gas chromatography retention times with the retention times of standard compounds and comparing mass spectral fragmentation patterns.

### 2.16 Statistical analysis

The two-way ANOVA analysis was conducted using MSTAT-C ver. 2.1. (Michigan State University, Michigan, USA). Mean comparisons were done *via* the least significant difference test (LSD) at 1% and 5% probability levels. Excel software was applied to draw graphs. Heat map cluster and Pearson correlation analysis were drawn using Rstudio ver. 14.2.1 software (github.com/talgalili/gplots).

## 3 Results

### 3.1 Arbuscular mycorrhizal fungi colonization

**Figure 1:**
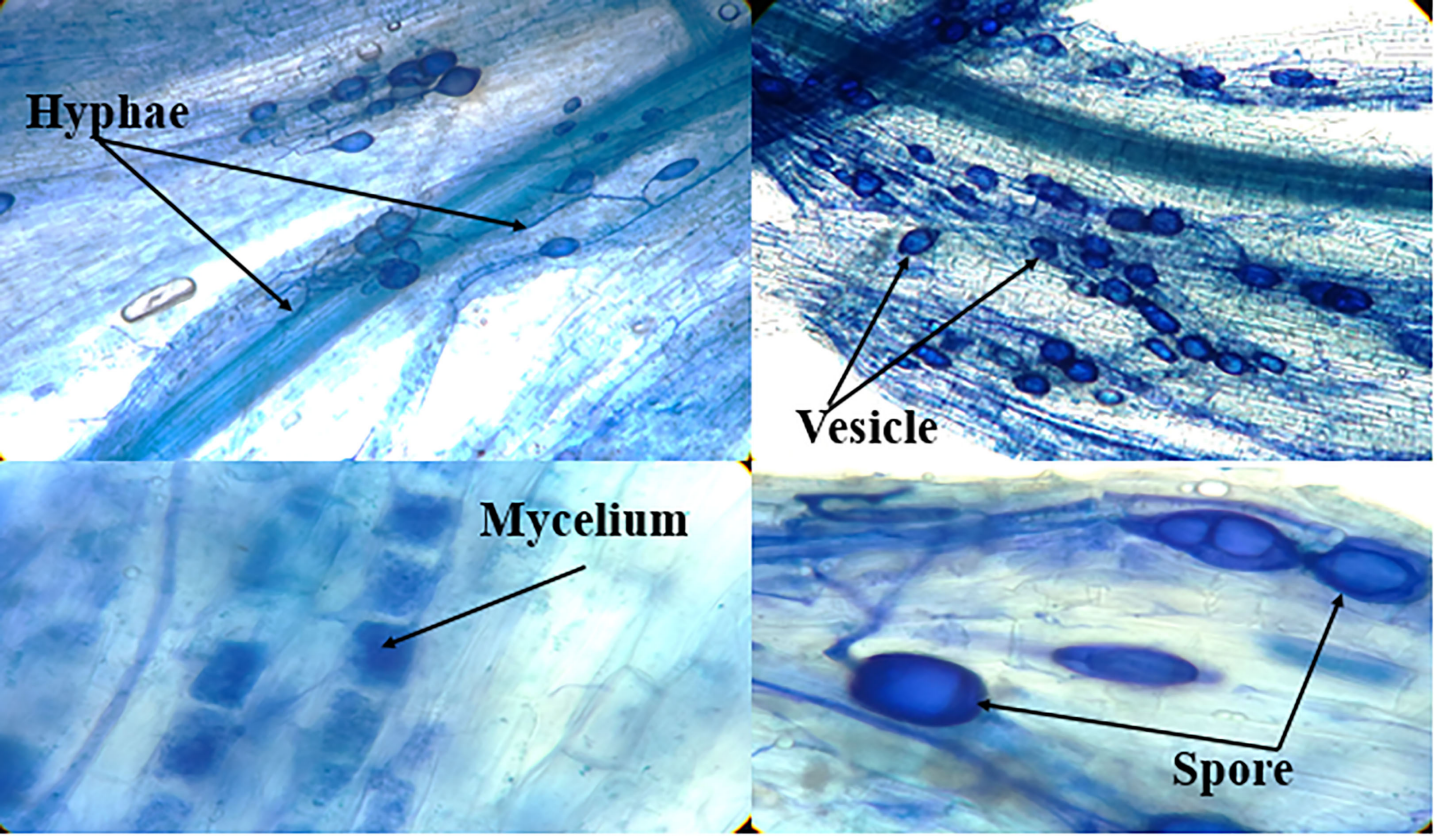
Microscopic images of the stained summer savory root fragments for detection of arbuscular mycorrhizae (Funneliformis mosseae) colonization.

Microscopic images of summer savory root fragments were traced to detect arbuscular mycorrhizal colonization, which is shown in **Figure 1**. High-quality photographs documented the AMF organs and hyphae that appear in blue.

### 3.2 Growth parameters

Drought stress and AMF inoculation significantly influenced some morphological traits of summer savory plants. Plant height and shoot mass (FW and DW) were significantly decreased up to 60, 78, and 80% respectively, under severe drought stress (30% filed capacity (FC)), as compared to the control plants (**Table 1**). The plant growth markers, plant height and shoot mass, were considerably enhanced in the plants treated with AMF inoculation (AMF_1_) relative to the control plants, up to 22, 15 and 21%. The highest assessment for all growth markers was documented when the plants were treated with AMF inoculation without drought stress.

**Table 1:** The effect of arbuscular mycorrhiza fungi (AMF) inoculation on growth parameters, RWC and MSI in summer savory plants under drought stress conditions.

Water stress	Inoculation status	Plant height (cm)	Shoot mass FW (g)	Shoot mass DW (g)	RWC (%)	MSI (%)
Control (100% FC)	AMF_0_	41.48 ± 1.35^b^	5.78 ± 0.26^b,c^	2.12 ± 0.10^c^	54.03 ± 0.25^b^	4.20 ± 0.37^b^
	AMF_1_	50.63 ± 0.55^a^	6.98 ± 0.02^a^	3.03 ± 0.03^a^	64.01 ± 0.75^a^	4.70 ± 0.17^a^
Moderate (60% FC)	AMF_0_	34.54 ± 0.27^c^	5.39 ± 0.03^b^	2.29 ± 0.03^bc^	47.83 ± 0.30^c^	3.76 ± 1.16^d^
	AMF_1_	39.54 ± 0.49^b^	6.56 ± 0.05^b^	2.44 ± 0.03^b^	52.91 ± 0.99^b^	4.14 ± 0.60^c^
Severe (30% FC)	AMF_0_	24.40 ± 0.28^e^	4.51 ± 0.04^d^	1.69 ± 0.4^d^	39.37 ± 0.45^e^	3.08 ± 1.28^e^
	AMF_1_	29.62 ± 0.38^d^	5.55 ± 0.02^c^	2.07 ± 0.02^c^	42.97 ± 0.37^d^	3.11 ± 0.94^e^
LSD at 0.05%		3.07	0.395	0.238	2.93	3.91
S.O.V.	df					
Drought	2	796.90^**^	4.399^**^	907.72^**^	1.368^**^	1.255^**^
AMF	1	290.28^**^	0.487^**^	312.60^**^	7.469^*^	1.737^**^
Drought × AMF	2	27.90^**^	0.146^**^	13.66^*^	4.062^**^	0.383^**^
Error	24	3.03	0.008	3.12	0.055	0.020
C.V.	–	3.47	4.30	4.81	3.86	6.25

FC, AMF0, and AMF1 refer to the filed capacity, AMF inoculation, without, and with. Different letters in columns indicate significantly different values at p ≤ 0.05. *, **, and ns, indicate significance at 5% and 1% probability levels and non-significant, respectively. AMF, FW, DW, RWC, MSI, S.O.V., C.V. and df refer to the arbuscular micorrhial fungi, fresh weight, dry weight, relative water content, membrane stability index, source of variation, coefficient of variation, and degree of freedom, respectively.

### 3.3 Leaf relative water content and membrane stability index

The leaf RWC and MSI have been decreased with increasing drought stress. Extreme drought stress (30% FC) significantly reduced RWC and MSI up to 64.8 and 52.6% compared to the control plants. AMF application again, similarly to growth parameters results, at both drought stress levels, improved the values of these two parameters compared to the non-inoculated ones (**Table 1**).

### 3.4 H_2_O_2_ and MDA content

Drought stress resulted in enhanced accumulation of H_2_O_2_ and MDA in summer savory plants, so the highest values for the traits were observed under the severe water deficit (30% FC), increased by 52.6% and 362%, compared to control plants, respectively. In contrast, the lowest H_2_O_2_ and MDA were documented in the plants under without drought stress. At severe drought stress; AMF inoculated plants had a decreased accumulation of H_2_O_2_ and MDA compared to the non-inoculated counterparts (**Table 2**).

**Table 2:** The effect of arbuscular mycorrhiza fungi (AMF) inoculation on H2O2, MDA, and proline content in summer savory plants under drought stress conditions.

Water stress	Inoculation status	H_2_O_2_ (µmol g^-1^ FW)	MDA (nmol g^−1^ FW)	Proline (µmol g^-1^ FW)
Control (100% FC)	AMF_0_	3.08 ± 0.03d	1.41 ± 0.10d	3.54 ± 0.30d
	AMF_1_	3.11 ± 0.03d	1.54 ± 0.10d	4.02 ± 0.24d
Moderate (60% FC)	AMF_0_	4.14 ± 0.04b	3.67 ± 0.06c	7.20 ± 0.04c
	AMF_1_	3.76 ± 0.05c	3.66 ± 0.05c	8.37 ± 0.07b
Severe (30% FC)	AMF_0_	4.70 ± 0.05a	6.52 ± 0.25a	11.89 ± 0.20a
	AMF_1_	4.20 ± 0.05b	5.28 ± 0.23b	8.69 ± 0.10b
LSD at 0.05%		0.150	0.558	0.690
S.O.V.	df			
Drought	2	4.399^**^	61.93^**^	107.72^**^
AMF	1	0.487^**^	12.70^**^	12.57^*^
Drought × AMF	2	0.146^**^	3.98^**^	8.47^**^
Error	24	0.008	0.11	0.16
C.V	–	4.30	6.99	5.63

FC, AMF0 and AMF1 refer to the filed capacity, AMF inoculation, without and with. Different letters in columns indicate significantly different values at p ≤ 0.05. *, **, and ns, indicate significance at 5% and 1% probability levels and non-significant, respectively. AMF, MDA, S.O.V., C.V. and df refer to the arbuscular mycorrhizal fungi, malondialdehyde, source of variation, coefficient of variation, and degree of freedom, respectively.

### 3.5 Proline content

Increased proline content was observed in response to moderate (60% FC) and severe (30% FC) drought stress (**Table 2**). The moderate drought-stressed plants with and without AMF had increased proline content compared to plants without AMF inoculation. Higher proline content in plants was recorded under severe drought stress, while it was reduced using AMF application (**Table 2**).

### 3.6 Total soluble proteins content

Drought stress significantly reduced the total soluble proteins (TSP) content of plants, while the trait was enhanced using AMF inoculation. AMF treatment improved the total soluble protein content by 343% compared to the severe (30%) drought-stress plants (**Table 3**). Moreover, the extreme drought stress caused a marked reduction in protein content over the control. In contrast, the moderate drought stress-induced reduction in protein content was nearly conquered by the AMF treatment. AMF treatment thus contributed to recovering the decline in the total protein content of summer savory plants under moderate and severe drought stress conditions.

**Table 3:** The effect of arbuscular mycorrhiza fungi (AMF) inoculation on the total protein content, antioxidant activity, and antioxidant enzyme activities in summer savory plants under drought stress conditions.

Water stress	Inoculation status	TSP (mg g^−1^ FW)	TAA (%)	APX (U mg^-1^ protein min^-1^)	GPX (U mg^-1^ protein min^-1^)	SOD (U mg^-1^ protein min^-1^)
Control (100% FC)	AMF_0_	3.83 ± 0.05b	4.31 ± 0.432e	0.210 ± 0.002c	2.13 ± 0.012e	0.65 ± 0.021e
	AMF_1_	4.66 ± 0.23a	5.10 ± 0.310ed	0.202 ± 0.008c	2.11 ± 0.014e	0.69 ± 0.022e
Moderate (60% FC)	AMF_0_	2.85 ± 0.05c	6.31 ± 0.303d	0.274 ± 0.003b	2.50 ± 0.013c	1.12 ± 0.007d
	AMF_1_	3.25 ± 0.05c	8.23 ± 0.321c	0.332 ± 0.004a	2.88 ± 0.020b	1.24 ± 0.014c
Severe (30% FC)	AMF_0_	1.05 ± 0.10e	9.50 ± 0.087b	0.316 ± 0.002ab	3.29 ± 0.016d	1.38 ± 0.008b
	AMF_1_	2.38 ± 0.06d	11.95 ± 0.224a	0.368 ± 0.004ba	3.43 ± 0.019a	1.52 ± 0.014a
LSD at 0.05%		0.423	1.27	0.053	0.047	0.075
S.O.V.	df					
Drought	2	16.00^**^	91.32^**^	0.049^**^	3.859^**^	1.574^**^
AMF	1	5.46^**^	10.59^**^	0.002^ns^	0.037^**^	0.001^ns^
Drought × AMF	2	0.55^**^	7.53^**^	0.008^**^	0.184^*^	0.048^**^
Error	24	0.063	0.59	0.001	0.001	0.002
C.V	–	8.32	9.20	4.60	3.32	4.16

FC, AMF0 and AMF1 refer to the filed capacity, AMF inoculation, without and with. Different letters in columns indicate significantly different values at p ≤ 0.05. *, **, and ns, indicate significance at 5% and 1% probability levels and non-significant, respectively. TSP, TAA, APX, GPX, SOD, AMF, S.O.V., C.V. and df refer to the total soluble protein, total antioxidants activity, ascorbate peroxidase, guaiacol peroxidase, superoxide dismutase, arbuscular mycorrhiza fungi, source of variation, coefficient of variation, and degree of freedom, respectively.

### 3.7 Total antioxidant activity

TAA of summer savory plants, measured by the FRAP method, was significantly influenced by drought stress and the application of AMF. The plants grown under severe drought stress with AMF inoculation showed the maximum TAA, which increased by 177% compared to the control. The AMF inoculation increased TAA values under ideal water conditions and drought stress. The summer savory plants grown under the conditions without water stress and AMF showed a minimum of TAA (**Table 3**).

### 3.8 Activity of antioxidant enzymes

The activity of antioxidant enzymes; APX, GPX, and SOD had the same trend in response to drought stress. As the stress intensified, the activity of all three enzymes significantly increased compared to the control. So, their enhancement was 71.4%, 61.0%, and 133.8%, respectively. All of the above-mentioned activities reached the maximum activity when the plants were subjected to severe stress (30% FC). The results showed that AMF inoculation together with severe and moderated drought conditions increased the activity of these enzymes. These enhancements by AMF application and severe water deficit were 16.5%, 4.3%, and 10.1% for APX, GPX, and SOD, respectively those compared to without AMF (**Table 3**).

### 3.9 Chlorophyll fluorescence parameters

With increasing drought stress, the *F_0_* parameter (minimum chlorophyll fluorescence yield in the light-adapted state) was enhanced, but other parameters including *Fm, Fv*, and *Fv/Fm* were reduced (**Table 4**). Drought stress raised the *F_0_* parameter by 45.8% and lessened *Fm, Fv*, and *Fv/Fm* by 38.6, 63.6, and 22.9%, compared to the control, respectively. However, AMF treatment alleviated the chlorophyll fluorescence parameters under moderate and severe drought stress conditions.

**Table 4:** The effect of arbuscular mycorrhiza fungi (AMF) inoculation on the chlorophyll fluorescence parameters in summer savory plants under drought stress conditions.

Water stress	Inoculation status	*F_0_*	*Fm*	*Fv*	*Fv/Fm*
Control (100% FC)	AMF_0_	0.554 ± 0.005c	3.22 ± 0.031^b^	2.67 ± 0.034^b^	0.828 ± 0.002ab
	AMF_1_	0.474 ± 0.014d	3.48 ± 0.018^a^	3.01 ± 0.030^a^	0.864 ± 0.004a
Moderate (60% FC)	AMF_0_	0.667 ± 0.010b	2.50 ± 0.020^e^	1.83 ± 0.020^d^	0.733 ± 0.001cd
	AMF_1_	0.633 ± 0.006b	2.95 ± 0.041^c^	2.31 ± 0.053^c^	0.785 ± 0.005bc
Severe (30% FC)	AMF_0_	0.808 ± 0.004a	2.72 ± 0.033^d^	1.84 ± 0.026^d^	0.703 ± 0.003d
	AMF_1_	0.669 ± 0.003b	2.51 ± 0.027^e^	1.91 ± 0.032^d^	0.733 ± 0.006cd
LSD at 0.05%		0.053	0.106	0.119	0.053
S.O.V.	df				
Drought	2	0.128^**^	1.591^**^	2.593^**^	0.043^**^
AMF	1	0.054^**^	0.207^**^	0.472^**^	0.012^**^
Drought ×AMF	2	0.007^**^	0.282^**^	0.203^**^	0.001^*^
Error	24	0.001	0.004	0.005	0.001
C.V	–	8.32	5.80	3.08	5.63

FC, AMF0 and AMF1 refer to the filed capacity, AMF inoculation, without and with. Different letters in columns indicate significantly different values at p ≤ 0.05. *, **, and ns, indicate significant at 5% and 1% probability levels and non-significant, respectively. F0, Fm, Fv, Fv/Fm, S.O.V., C.V., and df refer to the minimum amount of chlorophyll fluorescence, maximum amount of chlorophyll fluorescence, difference between F0 and Fm, maximum PSII quantum efficiency, source of variation, coefficient of variation, and degree of freedom, respectively.

### 3.10 Photosynthetic pigments

**Figure 2:**
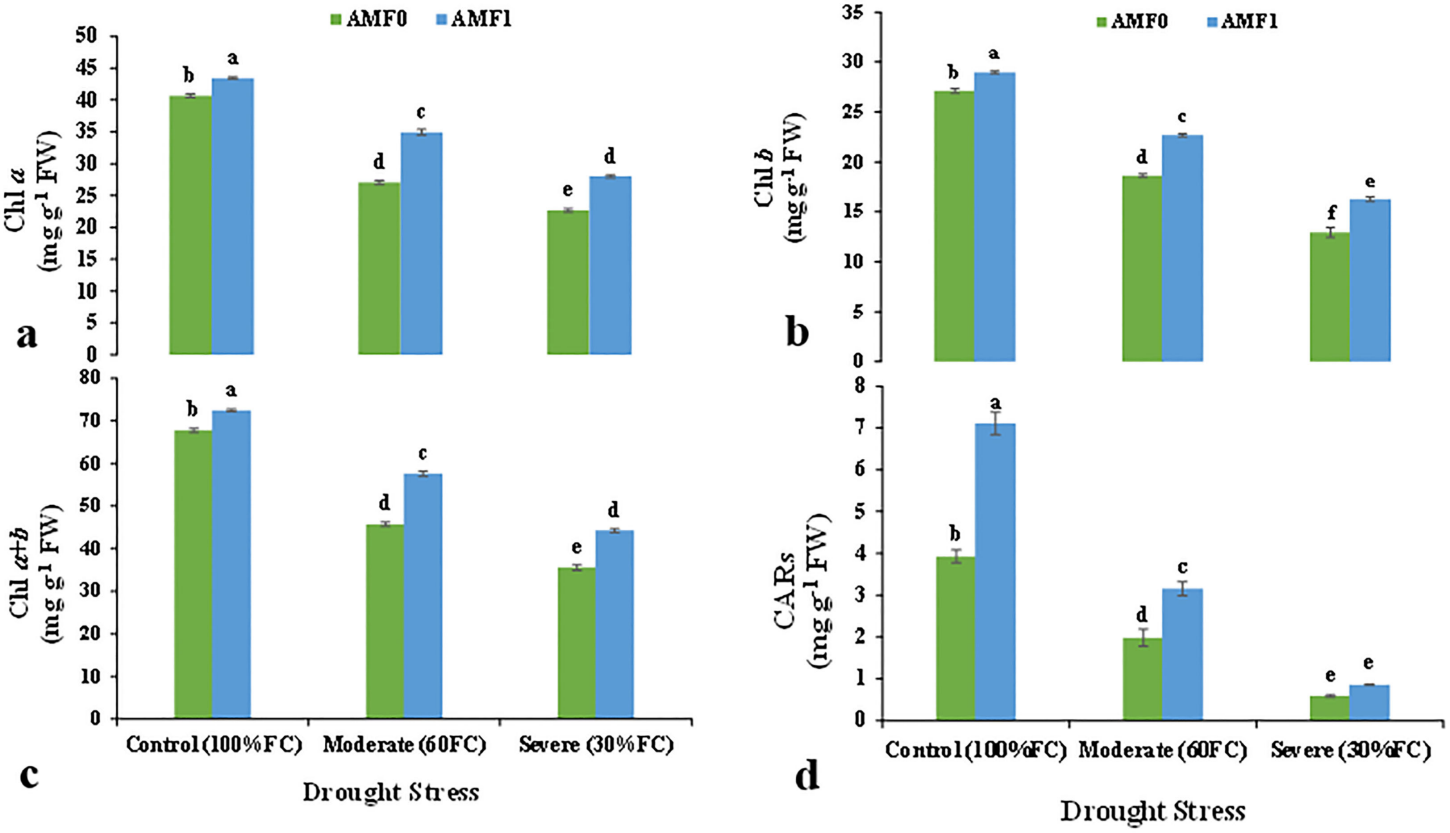
Effect of AMF inoculation on chlorophyll *a* (Chl *a*) **(A)**, chlorophyll *a* (Chl *b*) **(B)**, total chlorophyll (Chl *a+b*) **(C)**, and carotenoids (CARs) **(D)** the content in summer savory plants under drought stress conditions. Different letters indicate significant differences according to the LSD test at *p*<0.05. AMF0 refer to without AMF inoculation and AMF1 with AMF inoculation.

The photosynthetic pigments, chlorophylls Chl *a, b, a+b*, and carotenoids (CARs) content were significantly influenced by drought stress and AMF inoculation in the summer savory plants (**Table 5**). Specifically, under severe drought stress, the content of Chl *a*, *b*, *a+b*, and CARs decreased significantly by 40.0, 19.3, 26.9, and 213%, compared to the control plants, respectively (**Figures 2A–D**). AMF inoculation increased the content of the photosynthetic pigments. Moreover, AMF treatment retrieved Chl *a*, *b*, *a+b*, and CARs contents in the plants subjected to moderate and severe stress (**Figure 2**).

**Table 5:** Analysis of variance (ANOVA) for the effect of arbuscular mycorrhizal fungi (AMF) inoculation on the content of the photosynthetic pigments in summer savory plants under drought stress conditions.

S.O.V.	df	Chl *a*	Chl *b*	Chl *a+b*	CARs
Drought	2	724.24^**^	453.9^*^	2315.36	58.50^**^
AMF	1	213.55^**^	71.58^**^	532.56	17.78^**^
Drought ×AMF	2	16.16^**^	3.14^*^	33.26	5.52^**^
Error	24	0.976	0.770	3.15	0.193
C.V	–	3.01	5.16	6.30	12.96

S.O.V., df, chl, CARs, AMF, and C.V. refer to the source of variation, degree of freedom, chlorophyll, carotenoids, arbuscular mycorrhizal fungi, and coefficient of variation respectively. *, **, and ns, significant at the 5% and 1% probability levels and non-significant, respectively.

### 3.11 Essential oil composition

In the essential oil of *Satureja hortensis* L. plants, there were identified 27 constituents, accounting for 89.2-99.8% of the total EO (**Table 6**). The results revealed that carvacrol (50.84-60.03%) was the main ingredient of summer savory EO. In addition, γ-terpinene (19.03-27.33%), *p*-cymene (1.73-3.01%), α-terpinene (2.03-2.62%), myrcene (1.28-1.58%), and aromadendrene (1.10-1.52%) were identified as other predominant constituents of summer savory essential oil. The highest carvacrol and γ-terpinene contents, up to 57.03 and 24.33%, were obtained with AMF inoculation (AMF_1_). The lowest content of 50.84 and 19.03% was identified under severe drought stress (30% FC) without AMF inoculation. The highest *p*-cymene content (3.03%) was achieved under severe drought (30% FC) with AMF inoculation, and the lowest one (1.73%) was recorded for control plants. The top of the content of α-terpinene (2.63%) was observed under severe drought (30% FC) with AMF inoculation, and the lowest (2.03%) under moderate drought (60% FC) without AMF inoculation.

**Table 6:** The effect of the arbuscular mycorrhiza fungi (AMF) inoculation on essential oil (EO) composition of Satureja hortensis L. plants influenced by drought stress.

Treatments								
No	Component	RI literature	(100% FC) + AMF_0_	(100% FC) + AMF_1_	(60% FC) + AMF_0_	(60% FC) + AMF_1_	(30% FC) + AMF_0_	(30% FC) + AMF_1_
1	*α*-Thujene	924	0.74 ± 0.06	0.54 ± 0.14	0.48 ± 0.04	0.61 ± 0.03	0.52 ± 0.87	0.65 ± 0.08
2	*α*-Pinene	932	0.57 ± 0.02	0.47 ± 0.11	0.54 ± 0.06	0.4 ± 0.01	0.66 ± 0.11	0.57 ± 0.09
3	Camphene	946	0.2 ± 0.07	0.13 ± 0.03	0.18 ± 0.01	0.47 ± 0.02	0.21 ± 0.02	0.17 ± 0.02
4	*β*-Pinene	974	0.38 ± 0.01	0.22 ± 0.08	0.38 ± 0.10	0.32 ± 0.04	0.56 ± 0.03	0.49 ± 0.01
5	Myrcene	988	1.58 ± 0.11	1.28 ± 0.13	1.25 ± 0.04	1.39 ± 0.02	1.36 ± 0.06	1.46 ± 0.15
6	*n*-Decane	1000	0.5 ± 0.01	0.56 ± 0.08	0.52 ± 0.02	0.5 ± 0.09	0.56 ± 0.03	0.7 ± 0.05
7	*α-*Phellandrene	1002	0.27 ± 0.05	0.22 ± 0.04	0.47 ± 0.07	0.29 ± 0.02	0.33 ± 0.05	0.38 ± 0.11
8	*δ-3*-Carene	1008	0.01 ± 0.02	0.22 ± 0.04	0.25 ± 0.05	0.14 ± 0.04	0.14 ± 0.02	0.2 ± 0.01
9	*α*-Terpinene	1014	2.34 ± 0.19	2.53 ± 0.07	2.03 ± 0.21	2.27 ± 0.21	2.34 ± 0.03	2.62 ± 0.39
10	*p*-Cymene	1020	2.81 ± 0.12	1.73 ± 0.54	2.25 ± 0.44	2.83 ± 0.17	2.56 ± 0.12	3.01 ± 0.53
11	*β*-Phellandrene	1025	0.22 ± 0.02	0.12 ± 0.05	0.85 ± 0.06	0.34 ± 0.04	0.27 ± 0.04	0.27 ± 0.02
12	*(E)-β*-Ocimene	1044	0.14 ± 0.06	0.01 ± 0.04	0.26 ± 0.01	0.18 ± 0.02	0.18 ± 0.02	0.22 ± 0.03
13	γ-Terpinene	1054	20.16 ± 0.77	24.33 ± 2.27	22.12 ± 3.50	20.21 ± 0.52	19.03 ± 0.52	21.34 ± 0.17
14	*cis*-Sabinene hydrate	1065	0.09 ± 0.02	0.28 ± 0.05	0.35 ± 0.05	0.22 ± 0.02	0.27 ± 0.02	0.31 ± 0.09
15	*trans-*Sabinene hydrate	1098	0.08 ± 0.03	0.23 ± 0.09	0.28 ± 0.08	0.31 ± 0.04	0.35 ± 0.04	0.3 ± 0.01
16	Menthofuran	1159	0.06 ± 0.03	0.24 ± 0.06	0.7 ± 0.03	0.24 ± 0.11	0.34 ± 0.09	0.2 ± 0.01
17	Menthol	1167	0.08 ± 0.02	0.22 ± 0.04	0.17 ± 0.04	0.19 ± 0.03	0.21 ± 0.03	0.27 ± 0.02
18	Terpinen-4-ol	1174	0.28 ± 0.05	0.24 ± 0.05	0.18 ± 0.01	0.27 ± 0.02	0.25 ± 0.02	0.26 ± 0.03
19	*n*-Dodecane	1200	0.19 ± 0.02	0.21 ± 0.05	0.65 ± 0.01	0.17 ± 0.02	0.28 ± 0.02	0.17 ± 0.02
20	Thymol methyl ether	1232	0.18 ± 0.03	0.21 ± 0.21	0.38 ± 0.04	0.23 ± 0.03	0.55 ± 0.03	0.25 ± 0.02
21	Thymol	1289	0.27 ± 0.03	0.25 ± 0.05	0.35 ± 0.02	0.63 ± 0.13	0.18 ± 0.01	0.38 ± 0.45
22	Carvacrol	1298	53.17 ± 0.25	57.06 ± 1.49	57.02 ± 1.16	55.59 ± 1.20	50.84 ± 0.54	54.06 ± 4.44
23	Thymol acetate	1349	0.32 ± 0.01	0.2 ± 0.04	0.31 ± 0.00	0.17 ± 0.02	0.18 ± 0.03	0.26 ± 0.06
24	(*E*)-Caryophyllene	1417	0.29 ± 0.03	0.39 ± 0.03	0.27 ± 0.01	0.21 ± 0.04	0.23 ± 0.00	0.38 ± 0.03
25	Aromadendrene	1439	1.45 ± 0.11	1.16 ± 0.12	1.52 ± 0.06	1.15 ± 0.07	1.34 ± 0.00	1.10 ± 0.04
26	Viridiflorene	1496	0.19 ± 0.00	0.21 ± 0.04	0.36 ± 0.07	0.24 ± 0.06	0.34 ± 0.02	0.25 ± 0.05
27	*β*-Bisabolene	1505	0.25 ± 0.02	0.28 ± 0.07	0.48 ± 0.03	0.6 ± 0.02	0.25 ± 0.01	0.21 ± 0.04
22	Total Identified (%)		93.75	99.81	95.82	89.93	89.24	92.35

### 3.12 Pearson correlations, dendrogram clustering and principal component analysis

**Figure 3:**
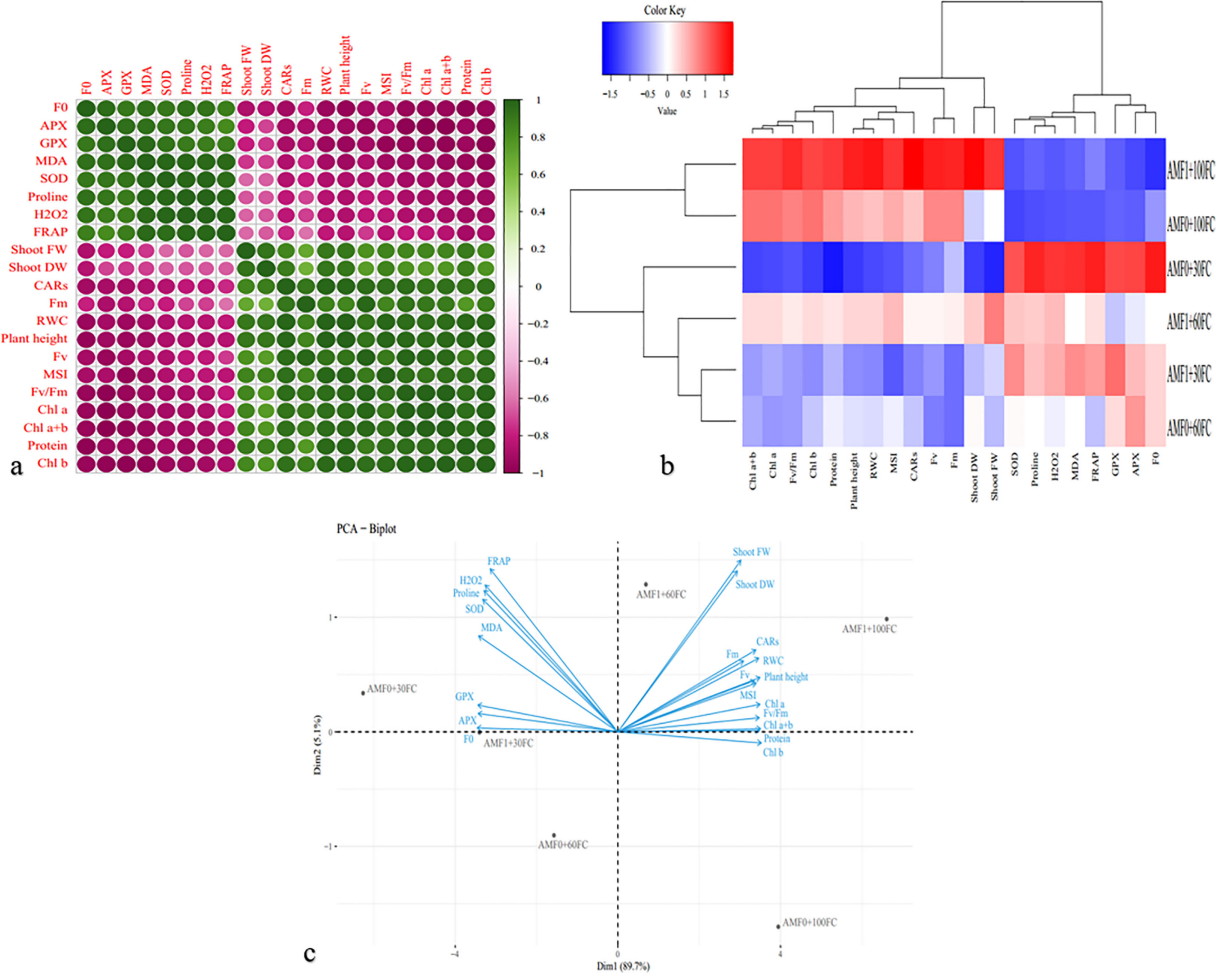
Heat map of Pearson correlations **(A)**, dendrogram clustering **(B)**, and principal component analysis PCA – Biplot **(C)** for the growth and biochemical responses of AMF inoculated summer savory plants exposed to drought stress conditions.

The correlation heat map exhibited a positive relationship among *F_0_*, antioxidant enzyme activities (APX, GPX, and SOD), total antioxidant activity (TAA, FRAP), the content of proline, and H_2_O_2_. Also, it was observed a strong positive correlation among growth parameters (shoot mass (FW and DW), and plant height), photosynthetic pigments (Chl *a*, Chl *b*, Chl *a+b*, and CARs contents), fluorescence parameters (*Fm*, *Fv*, *Fv/Fm*), RWC and MSI, and total soluble proteins content (TSP, protein). Finally, the results determined a significant negative correlation between the two mentioned groups above (**Figure 3A**). The dendrogram clustering heatmap analysis showed that the evaluated traits were classified into two clusters; cluster 1 contained *F_0_*, APX, GPX, SOD, total antioxidant activity, proline, and H_2_O_2_, and group 2 consisted of shoot mass (FW and DW), plant height, Chl *a*, Chl *b*, Chl *a+b*, CARs, *Fm*, *Fv*, *Fv/Fm*, RWC, MSI, and TSP, which confirmed the Pearson correlation. On the other hand, the plants subjected to these experimental treatments revealed two groups, as group 1 included the plants grown under regular irrigation along with AMF_0_ and AMF_1_. Group 2 had the plants grown under 60% FC without AMF inoculation and with AMF, and 30% FC without AMF inoculation and with AMF (**Figure 3B**). The PCA biplot showed that *F_0_*, APX, GPX, SOD, total antioxidant activity, proline, and H_2_O_2_ were associated with the severe drought stress condition without AMF inoculation and with AMF, while the traits such as shoot mass (FW, DW), plant height, Chl *a*, Chl *b*, Chl *a+b*, CARs, *Fm*, *Fv*, *Fv/Fm*, RWC, MSI, and TSP were linked to AMF inoculation under 100% FC and 60% FC (**Figure 3C**).

## 4 Discussion

Water deficit is an annoying stress factor at the global level, which excessively reduces agricultural crop production. Then, the effective methodologies for reducing the adverse water deficit effect should be considered. Over the past decades, researchers have focused on implementing and validating many tools and techniques to overcome drought stress (Begum et al., 2019). In the present study, drought stress decreased the growth characteristics, including the fresh and dry biomass and the height of the savory plant. However, AMF inoculation significantly compensated for the growth and yield reduction of the savory plant under drought stress. growth promotion, enhanced minerals, and water absorption in *Sophora davidii* seedlings under water stress is facilitated by the mycorrhizal hyphae expansion (Gong et al., 2013) which is in agreement with our results in summer savory plants. in the other study on coriander, AMF inoculation enhances drought tolerance by increasing water, nitrogen, phosphorus, and potassium absorption through its external hyphae, as a result, AMF-treated plants produce high biomass (Oliveira et al., 2016). In other words, through mycorrhizal hyphae, this fungus can absorb the nutrients that generally have little mobility and hardly enter the plant’s roots and increase the absorption of these nutrients by increasing the root surface and reducing the diffusion distance (Jaleel et al., 2007). In the same case, the growth and tolerance of tomato plants under drought stress were improved by inoculation with AMF (Chitarra et al., 2016) and the current findings are attuned with the mentioned reports above.

Our results showed that colonization behavior was improved by AMF inoculation in savory plants, which agrees with the results of Huang et al. (Huang et al., 2017). AMF inoculum develops from an infection point to the soil, and subsequently, secondary mycelium is formed, which can connect plants to the mycorrhizal network and finally establish colonization (Simard et al., 2012). Although there is a two-way flow of water in the hyphae, the possibility of water returning from the hyphae to the soil is much less, and the maximum water is directed from the AMF hyphae to the transpiration pathway in plants (Wu and Zou, 2009; Wu et al., 2013) that enhance the ability of water absorption by plants roots in water deficit. Besides, AMF colonization has an inverse relationship with the amount of ROS accumulation which decreases in the AMF-treated plants (Wu and Zou, 2009); most AMF species have the *Sod1* gene, which increases drought resistance and growth in such conditions. These genes are regulated with high molecular diversity inside the root and improve drought tolerance to some extent (Corradi et al., 2009).

The light energy absorbed by chlorophyll molecules is employed to drive photosynthesis in chloroplast apparatus and photochemical fluorescence centers, or it is dissipated as heat (Qiu et al., 2013), which depends on different biochemical and environmental conditions (Sheng et al., 2008). Chlorophyll fluorescence reflects the photosynthetic activity of leaves in a complex manner and is often used to analyze photosynthesis and related mechanisms in plants subjected to biotic or abiotic stressors (Qiu et al., 2013). The *F_v_/F_m_* ratio can be used to measure the initial photochemical capacity of photosystem II (PSII) (Sheng et al., 2008). Under AMF colonization, the improvement of water status and stomatal opening in AMF-inoculated plants can increase the efficiency of PSII (*F_v_/F_m_*) under drought-stress conditions. In the present study, AMF treatment mitigated the *F_v_/F_m_* in savory plants under drought stress. This indicates a symbiotic relationship between plants and AMF, which can increase chloroplast’s absorption efficiency and excitation energy and improve the photochemical capacity of PSII in leaves (Gong et al., 2013). Our data support the findings of previous studies showing that AMF symbiosis increased *F_v_/F_m_* in maize plants (Sheng et al., 2008; Zhu et al., 2012). Similarly, in another experiment under drought stress conditions, AMF inoculation improved the maximum quantum yield in *Ceratonia siliqua* L. (Boutasknit et al., 2021). The results show that AMF treatment under drought-stress conditions can enhance the natural use of light energy in the photochemical processes of plants (Boutasknit et al., 2020). In agreement with our findings, many studies have shown that AMF inoculation significantly increased the chlorophyll content of plants (Sannazzaro et al., 2006; Sheng et al., 2008).

AMF inoculation under stress conditions improves the chloroplast cycle and protects pigments from damage caused by photosystem reaction centers and stress by increasing energy absorption efficiency (Bagheri et al., 2011; Bashir et al., 2020). On the other hand, the accumulation of osmolytes and minerals required for photosynthesis, as a result of AMF inoculation, partially increases the overall content of chlorophyll and improves the absorption of water and nutrients under drought stress (Abbaspour et al., 2012). Further, this action increases photosynthesis and biosynthesis and accumulation of protein and carbohydrate (Zaferanchi et al., 2019) which is in agreement with our findings on savory plants. Also, in the present experiment, the role of AMF in reducing the negative effect of drought stress was shown by improving the content of chlorophyll and photosynthetic pigments, which was consistent with the results of Boutasknit et al. (Boutasknit et al., 2021). Our result showed that the higher content of CARs in savory plants inoculated with AMF compared to those without AMF can also reflect the defensive reaction of plants related to higher TAA in normal and water stress conditions.

In addition to the limited cell division and reduced growth, loss of turgor is another consequence of drought stress, causing a decrease in maintaining high RWC in the plants, which is an essential factor in plant tolerance to drought stress. RWC and MSI have been introduced as the tolerance indicators of drought stress, and increasing their amount will improve the adverse conditions caused by stress (Bangar et al., 2019) which the reduction of these traits revealed mitigation of drought stress adverse effects. In the present study, despite the decrease in RWC and MSI due to drought stress, the RWC in AMF-inoculated plants improved compared to the control and is alleviated under moderate and severe drought stress. Maintaining cell mass and water relations in plants in response to water deficit largely depends on managing plant cells’ osmotically active molecules and ions (Farooq et al., 2009). While drought stress disrupts the coordination between transpiration and water uptake by plants and extensively reduces RWC; AMF mycelia help to absorb more water and nutrients (Ye et al., 2019) thus it helps to reduce RWC and MSI under water stress.

Our experiment showed that the AMF-treated plants possessed higher SOD, APX, and GPX activities under moderate stress conditions than plants without AMF inoculation, indicating that drought stress is often associated with high levels of reactive oxygen species generation (Fouad et al., 2014). In other words, in all plants, the most common technique to protect plants against oxidative stress damage is stimulating the biosynthesis of antioxidant enzymes such as SOD, APX, and GPX to reduce or eliminate ROS molecules (Mirzai et al., 2013). APX by using ascorbate as an electron donor converts H_2_O_2_ into H_2_O, which plays an essential role in protecting cells from the harmful effects of H_2_O_2_ and indicates the impact of AMF on the activity of the enzymes, as mentioned above. The results of this experiment were consistent with the findings of Begum et al. (Begum et al., 2022), on tobacco plants. Also, the higher activity of SOD in AMF-inoculated plants indicates that these plants have more efficient superoxide transformation, so more effective detoxification of superoxide radicals to H_2_O_2_ is done (Zou et al., 2015). Furthermore, AMF may initiate gene activation by utilizing ion and signal transfer, which can increase the expression of enzymes such as SOD under drought stress by absorbing some micronutrients such as Zn and Cu (Huang et al., 2017).

The present findings showed that plants exposed to drought stress especially severe water deficit treated with AMF showed a decrease in the accumulation of MDA and H_2_O_2_, which indicated less oxidative stress damage in plant cells compared to plants without AMF inoculation under water deficit stress. Also, the accumulation of H_2_O_2_ and MDA in plant tissues are among those that indicate the levels of oxidative stress damage to the cell membrane (Hossain et al., 2015). The decrease in MDA and H_2_O_2_ accumulation due to AMF application indicates the reduced lipid peroxidation rate in plants inoculated with AMF compared to without AMF, which was also clearly evident in our results. Aalipour et al. (Aalipour et al., 2020) in *Arizona cypress* plants reported a relatively lower accumulation of H_2_O_2_ and MDA in plants subjected to AMF. In addition, the lower rate of lipid peroxidation in plants accompanied by AMF indicates the increased tolerance to oxidative damage due to the enhanced activity of antioxidant enzymes, which is the most common mechanism to reduce the effect of ROS under drought stress (Talaat and Shawky, 2014). APX and GPX are also considered potential non-enzymatic antioxidants in the glutathione ascorbate cycle and play an important role in protecting plants against oxidative stress (Wu et al., 2006). The increase in the activity of these enzymes can also be a reflection of AMF inoculation in this study, which agrees with the experiment of Mirshad and Puthur (Mirshad and Puthur, 2016).

With any increase in the drought stress duration and intensity, and along with the enhancement in the activity of antioxidant enzymes, the amount of proline and other osmolytes also increases. In our research, those compound amounts were enhanced with the use of AMF in water deficit condition compared to control especially in moderate drought stress that has exhibited the importance of AMF role in enhancement of drought stress tolerance. Under certain conditions, some plants produce large quantities of proline to promote osmosis and prevent dehydration (Lu et al., 2007). These organic solutes, which act as osmotic buffers, facilitate water absorption to stabilize intracellular structures and protect them against the harmful effects of ROS (Wu et al., 2013). The increment in proline accumulation in the plants treated with AMF leads to a higher water potential gradient overcoming the osmotic imbalance. In addition to osmotic regulation, proline plays a vital role in protecting the enzymes involved in chlorophyll synthesis. Proline also reduces photodamage in the thylakoid membrane (Kishor et al., 2005). On the other hand, proline acts as a carbon and nitrogen reservoir, and its higher concentration in AMF-inoculated plants may provide energy for plant growth under drought stress (Hare and Cress, 1997). In other words, the accumulation of proline in the root can provide an osmotic mechanism for the plant to maintain a favorable potential gradient for water to enter the root, which leads to less stress damage in the plants (Porcel and Ruiz-Lozano, 2004). Abbaspour et al. (Abbaspour et al., 2012) reported similar results to the current findings in *Pistacia vera* L. In the present study, the AMF-inoculated savory plants had a higher content of soluble proteins under drought stress, which indicates an increase in the accumulation of proteins and their maintenance by the presence of AMF under drought stress. This action happens through the synthesis of osmolytes, whose catabolism is reduced by AMF inoculation, and thus their accumulation increases. In other words, AMF-mediated accumulation of proline, sugars, and glycine betaine may protect plants against ROS-induced oxidative damage to proteins and membranes, thereby improving protein levels (Hashem et al., 2016). The results of the present experiment were consistent with Begum et al. (Begum et al., 2019) on tobacco plants.

The results also demonstrated that the savory EO components’ percentages were enhanced under moderate drought stress. The increase in EO content under moderate drought stress conditions is one of the primary defense mechanisms in medicinal and aromatic plants, in this condition, the photosynthetic rate decreases due to the reduction in CO_2_ uptake (as a result of closing stomata), leading to the generation of NADPH^+^ (Sheteiwy et al., 2021). The increase in the compound is an inhibitory factor for the photosynthetic rate and plant productivity. Therefore, the productivity of secondary metabolites such as EO compounds, alkaloids, etc., as a function of the increase in NADPH^+^ consumption (accumulated in plant cells) can effectively decrease the negative impacts of drought stress in medicinal and aromatic plants (García-Caparrós et al., 2019).

Furthermore, the AMF treatment significantly increased the main constituents’ percentages under without, moderate, and severe drought stress. It seems that the application of AMF under normal and water stress circumstance improves nutrient accessibility and stimulates higher metabolic efficiency (e.g., nutrient transport), which have an essential role in the production of carbohydrates and the development of the glandular trichomes, EO channels, and secretory ducts. Additionally, AMF helps to convert inorganic N into organic N in the form of protein and chlorophyll, which leads to an increase in the photosynthetic rate. Zhao et al. (Zhao et al., 2022) noted the increase in primary photosynthesis compounds, including erythrose‐4‐phosphate, phosphoenolpyruvate, pyruvate, and glyceraldehyde‐3‐phosphate which play the leading role in increasing terpene constituents and EO productivity in medicinal and aromatic plants which it could be the reason of the EO constituents of summer savory plants improvement using AMF under normal and water stress and as a result, it led to an amelioration of water stress. In addition, Hazzoumi et al. (Hazzoumi et al., 2017) concluded that AMF inoculation stimulates the productivity of EO glands probably by increasing endogenous hormone levels, particularly cytokinin, indole‐3‐acetic acid, and gibberellin. Similarly, Hazzoumi et al. (Hazzoumi et al., 2015 **)** noted that the EO components of *Ocimum basilicum* were enhanced under moderate and severe drought stress conditions.

## 5 Conclusions

Drought stress declined the values of morphological traits, RWC, TSP, *Fm*, *Fv*, *Fv/Fm*, and photosynthesis pigments in the summer savory plants, while AMF inoculation alleviated those traits in control plants, and also in plants under moderate and severe drought stress. The results showed that water stress increased H_2_O_2_, MDA, and proline contents, and APX, SOD, and GPX activities, otherwise, AMF inoculation reduced the values of these attributes. TAA values were enhanced in the savory plants exposed to water stress and without/with AMF inoculation. Overall, the findings suggest that AMF inoculation enhanced the drought tolerance of the summer savory plants by improving the antioxidant defense system and could be a hopeful approach for ameliorating the damages caused by drought stress. However, there may be a need for more in-depth studies with other AMF species or a combination of them, especially in the field conditions to decide on their actual efficiency under drought conditions.

## Data availability statement

The raw data supporting the conclusions of this article will be made available by the authors, without undue reservation.

## Author contributions

Conceptualization: FR, MA and TA. Formal analysis: MA. Investigation and methodology: MA and FR. Visualization: TA and MA. Writing-original draft: TA, MH, and MA. Review and editing: SE, SS, MB, MM, and JM. All authors have read and agreed to the published version of the manuscript.
